# Prognostic factors associated with survival in patients with anaplastic oligodendroglioma

**DOI:** 10.1371/journal.pone.0211513

**Published:** 2019-01-30

**Authors:** Shuo Liu, Xiaoqiang Liu, Yingxiu Xiao, Shuying Chen, Weiduan Zhuang

**Affiliations:** Neurology Department, First Affiliated Hospital of Shantou University Medical College, Shantou, Guangdong, China; Bilkent University, TURKEY

## Abstract

Anaplastic oligodendroglioma is a rare disease with an inadequately understood prognosis. The aim of this study was to investigate factors associated with survival outcome in anaplastic oligodendroglioma patients. A population-based cohort study was conducted based on the Surveillance, Epidemiology, and End Results program. In total, 1899 patients with a histological diagnosis of anaplastic oligodendroglioma from 1973 to 2015 were included. Mean age at diagnosis was 49.2 years, and 56.19% were male. In our study, 62.40% of patients were married, and 87.05% were white. Most patients (90.42%) were diagnosed with anaplastic oligodendroglioma as their first malignant primary tumor, but 9.58% had a diagnosis of at least one other primary malignancy; 87.89% of patients had received cancer-directed surgery. Patients receiving surgery had a better prognosis for overall survival compared to those not receiving surgery after propensity score matching analysis (p<0.05). The overall 1-, 3-, 5-, and 10-year survival of anaplastic oligodendroglioma was 78.7%, 60%, 50.2%, and 36.2%, respectively. Kaplan-Meier analysis indicated that age, marital status, presence of multiple primary malignancies, and surgical treatment were associated with overall survival, whereas sex and race were not. Moreover, age at diagnosis of 52 years was calculated as an optimal cutoff value to distinguish better and worse overall survival. Multivariate Cox proportional hazard analysis indicated that older age (OR 1.040, 95%CI1.035–1.045), single patients (OR 1.293, 95%CI 1.103–1.515), and presence of multiple primary malignancies (OR 1.501, 95%CI 1.238–1.820) were significantly associated with worse overall survival, whereas surgery (OR 0.584, 95%CI 0.494–0.689) was associated with better overall survival. A nomogram predicting 5-, and 10-year survival probability for anaplastic oligodendroglioma was constructed based on these variables. In conclusion, age, marital status, presence of multiple primary malignancies, and surgical treatment were associated with survival of anaplastic oligodendroglioma.

## Introduction

Primary central nervous system (CNS) tumors are a diverse group of neoplasms arising from a wide range of CNS cells. They predominantly comprise gliomas.[1] As a group, oligodendrogliomas comprise the third most common type of primary glioma, accounting for 2% to 5% of all primary CNS tumors and 4% to 15% of gliomas.[2] Oligodendrogliomas are tumors of oligodendrocytes and often occur in patients aged 40 to 60 years, with an average age at diagnosis of approximately 45 years.[3,4] The World Health Organization (WHO) has divided oligodendroglioma into low-grade well-differentiated oligodendroglioma (WHO grade II) and anaplastic astrocytoma (AO) (WHO grade III).[4]

AO accounts for a small proportion of oligodendrogliomas with approximately 400 new cases predicted for 2016.[5] They either appear as a novel tumor or develop from low-grade oligodendrogliomas through anaplastic transformation. The incidence of AO peaks at age 55–64.[5] Clinical symptoms and signs of AO vary depending on the tumor location and do not dependably differentiate AO from other kinds of gliomas. In most clinical cases, seizures are the most common presenting symptom of AO. Other common symptoms include headaches, nausea, mental status change or weakness.[2,6]

Surgical treatment has been reported to be a favorable prognostic factor for oligodendroglioma[1] and is recognized as the keystone of treatment.[7,8] However, these conclusions are based on studies of both low-grade oligodendroglioma and AO, not on studies of AO alone. At present, there is minimal scientific literature assessing survival-related prognostic factors in AO patients. We therefore performed an analysis of AO patients in the population-based Surveillance, Epidemiology, and End Results (SEER) database. We intended to investigate the demographic characteristics of AO patients and discover factors associated with survival outcome.

## Materials and methods

### Study design and patient population

This study was conducted from data in the SEER program of the National Cancer Institute. This program is the largest publicly available cancer registry, which has been prospectively collecting cancer incidence, clinicopathological characteristics, and survival data from approximately 30% of the American population since 1973.[9] Every effort has been made to exclude identifying information on individual patients. We utilized the latest release data from the 2017 submission of the SEER database (1973–2015 data). Patients with a diagnosis of AO were selected from the SEER database using the International Classification of Diseases for Oncology, Third Edition (ICD-O-3) histology code 9451.

Clinical variables including age, sex, race, marital status, presence of multiple primary malignancies, surgical treatment, survival status, and survival time were collected. Patients with unknown data were excluded. Marital status at diagnosis was classified as single, married, or separated/divorced/widowed. Race was classified as white, black, or other. Presence of multiple primary malignancies was noted to differentiate patients with only AO from those with AO in addition to at least one other malignant primary tumor. Tumors not reported in the SEER database were assumed malignant. The primary outcome was the disease-specific overall survival.

### Statistical analyses

Data were summarized as mean ± standard deviation (sd) for continuous variables and as percentages for categorical variables. Categorical variables among different groups of patients were compared by Chi-squared test. A propensity score matching (PSM) analysis was utilized to adjust for baseline confounding factors. The PSM model was based upon age, sex, race, marital status, and presence of multiple primary malignancies. Overall survival (OS) was compared between the subgroups using Kaplan-Meier survival curves with log-rank test and univariate Cox proportional hazards analysis. Possible prognostic variables from univariate Cox proportional hazards analyses and Kaplan-Meier survival curves were admitted in a multivariate Cox proportional hazards analysis to assess which prognostic factors were independently associated with disease-specific OS. A nomogram was formulated based on the significant prognostic factors from the multivariate Cox proportional hazards analysis to obtain predicted survival probabilities at 5 and 10 years. Statistical analyses were performed by R software (3.5.0 version). A two-tailed p-value ≤0.05 was considered significant.

## Results

### Patient population and baseline characteristics

**Fig 1:**
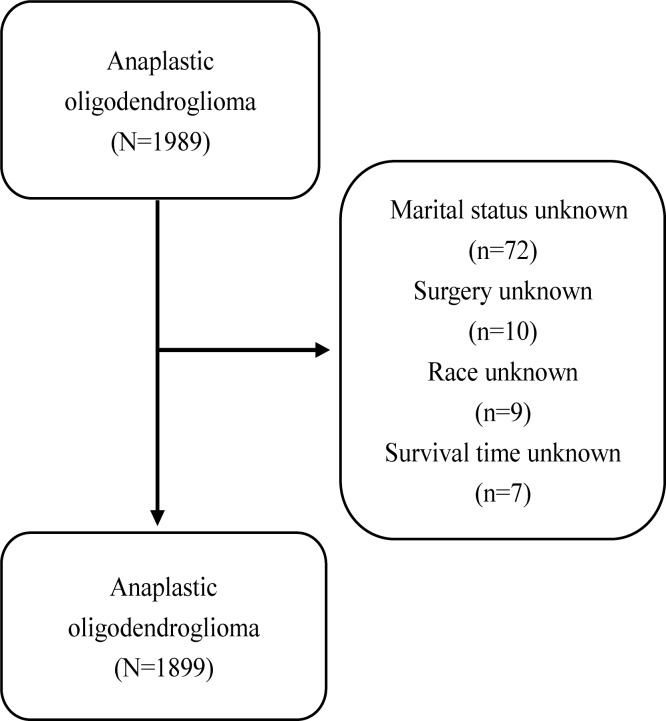
Flow diagram. A flow diagram of patient selection for the study cohort.

From 1973 to 2015, 1989 patients were diagnosed with AO. Patients with unknown information on any of the collected variables, including marital status (n = 72), race (n = 9), surgery (n = 10), and survival time (n = 7) were excluded. A total of 1899 patients were included for final analysis **(Table 1)**. The flow diagram is shown in **Fig 1**. The mean age at diagnosis was 49.2 years and 56.19% were male. 87.05% of patients were white, and 4.58% were black. The majority of patients were married (62.40%). Most patients (90.42%) were diagnosed with AO as their first malignant primary tumor, but 9.58% also had a diagnosis of at least one other primary malignancy. At the time of last follow-up, the mean survival time was 56.0 months and 44.81% patients were alive.

**Table 1:** Characteristics of patients with anaplastic oligodendroglioma.

Characteristics		Number
Number		1899
Age (year) mean±sd		49.2±15.4
Sex	Male	1067(56.19%)
	Female	832(43.81%)
Race	White	1653(87.05%)
	Black	87(4.58%)
	Others	159(8.37%)
Marital status	Single	432(22.75%)
	Married	1185(62.40%)
	Separated/divorced/widowed	282(14.85%)
Multiple primary malignancies	No	1717(90.42%)
	Yes	182(9.58%)
Surgery	Surgery	1669(87.89%)
	No Surgery	230(12.11%)
Survival status	Alive	851(44.81%)
	Dead	1048(55.19%)
Survival time (month) mean±sd		56.0±58.3

### Patient treatment

87.89% of patients received cancer-directed surgery. As shown in **Table 2**, patients who received surgical treatment were more likely to be younger. Multivariate logistic analysis showed this difference to be significant (p = 6.74e^-5^). However, there were no significant differences in sex, race, marital status or presence of multiple primary malignancies between patients who received surgery or not.

**Table 2:** Patient characteristics stratified by surgery.

Characteristics		Surgery N = 1669	No surgery N = 230	p-value
Age (year)		48.68±14.93	53.00±17.97	<0.001
Sex	Male	925 (55.4%)	142 (61.7%)	0.082
	Female	744 (44.6%)	88 (38.3%)	
Race	White	1452 (87.0%)	201 (87.4%)	0.765
	Black	75 (4.5%)	12 (5.2%)	
	Others	142 (8.5%)	17 (7.4%)	
Marital status	Single	380 (22.8%)	52 (22.6%)	0.851
	Married	1044 (62.6%)	141 (61.3%)	
	Separated/divorced/widowed	245 (14.7%)	37 (16.1%)	
Multiple primary malignancies	No	1514 (90.7%)	203 (88.3%)	0.281
	Yes	155 (9.3%)	27 (11.7%)	
Survival status	Alive	789 (47.3%)	62 (27.0%)	<0.001
	Dead	880 (52.7%)	168 (73.0%)	
Survival time (month) mean±sd		57.89±58.04	42.27±58.47	<0.001

PSM was utilized in comparing the surgical and non-surgical treatment groups to adjust for the impact of confounders. Surgical patients were matched 1:1 with non-surgical patients, based on age, sex, race, marital status, and multiple primary malignancies. If multiple best matches existed, only one was utilized. After PSM, 230 pairs of patients were matched and compared. We found that surgical patients had a better prognosis for OS compared to non-surgical (P = 0.006). Furthermore, multivariate Cox regression analysis demonstrated that surgical patients had a better prognosis for OS, even after PSM. (p<0.001).

### Survival

**Fig 2:**
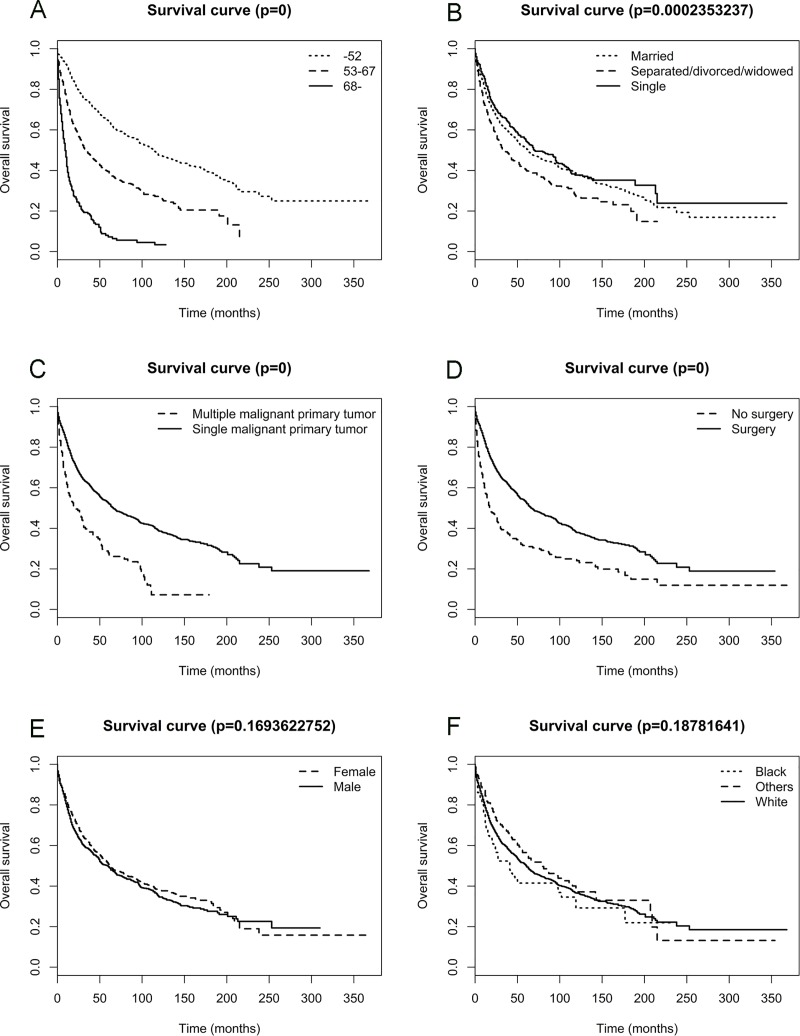
Survival analysis by risk factors. (A) Survival curve of age and patient survival in months; (B) Survival curve of marital status and patient survival in months; (C) Survival curve of multiple primary malignancies and patient survival in months; (D) Survival curve of surgical treatment and patient survival in months; (E) Survival curve of sex and patient survival in months; (F) Survival curve of race and patient survival in months.

The overall 1-, 3-, 5-, and 10-year survival was 78.7%, 60%, 50.2%, and 36.2%, respectively. Kaplan-Meier curves indicated that age **(Fig 2A)**, marital status **(Fig 2B)**, multiple primary malignancies **(Fig 2C)**, and surgical treatment **(Fig 2D)** were associated with OS whereas sex **(Fig 2E)**, and race **(Fig 2F)** were not. Moreover, the age of 52 years was calculated as an optimal cutoff value to distinguish between better and worse OS. At time of diagnosis, patients older than 52 years had 1.37 times the risk of worse OS than those younger (P<0.05). In **Table 3**, all variables were included in a univariate Cox proportional hazards analysis. These results were coherent with the outcomes of Kaplan-Meier analysis (all p<0.05). Significant variables from univariate analyses and Kaplan-Meier survival curves were the further admitted into a multivariate Cox proportional hazards analysis. Older age (OR 1.040, 95%CI 1.035–1.045), single patients (OR 1.293, 95%CI 1.103–1.515), and presence of multiple primary malignancies (OR 1.501, 95%CI 1.238–1.820) were significantly associated with worse OS whereas surgical treatment (OR 0.584, 95%CI 0.494–0.689) was associated with better OS.

**Table 3:** Univariate and multivariate Cox proportional hazard analyses of clinical characteristics for overall survival in patients with anaplastic oligodendroglioma.

Factor	Category	Univariate		Multivariate	
		HR (95% CI)	p-value	HR (95% CI)	p-value
Age	-	1.041 (1.036–1.045)	<2e^-16^	1.040 (1.035–1.045)	<2e^-16^
Sex	Female	Reference	-	-	-
	Male	1.09 (0.964–1.232)	0.169	-	-
Race	Black	Reference	-	-	
	White	0.833 (0.628–1.104)	0.204	-	-
	Others	0.722 (0.509–1.025)	0.068	-	-
Marital Status	Married	Reference	-	-	-
	Single	0.895 (0.768–1.043)	0.155	1.293 (1.103–1.515)	0.002
	Separated/divorced/ Widowed	1.326 (1.126–1.562)	0.001	1.060 (0.897–1.252)	0.494
Multiple primary malignancies	No	Reference	-	-	-
	Yes	2.069 (1.716–2.496)	2.7e^-14^	1.501 (1.238–1.820)	3.5e^-5^
Surgery	No surgery	Reference	-	-	-
	Surgery	0.543 (0.460–0.641)	4.4e^-13^	0.584 (0.494–0.689)	2.1e^-10^

### Nomograms

**Fig 3:**
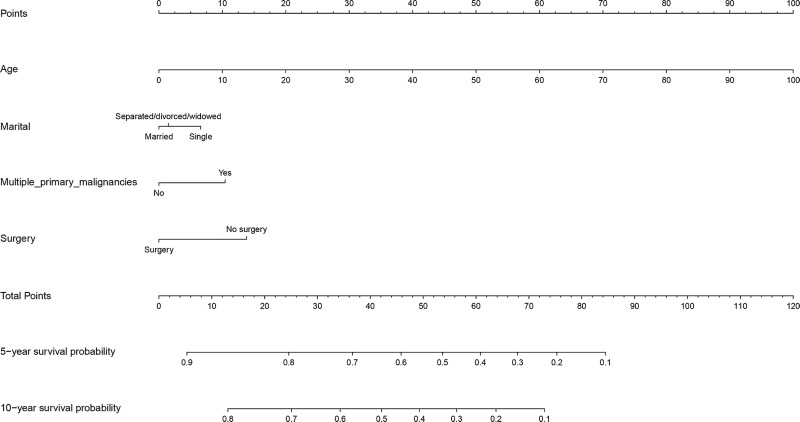
Nomogram for predicting 5- and 10-year survival probability of anaplastic oligodendroglioma.

The independent predictors from the multiple Cox proportional hazards analysis were used to create a nomogram for predicting survival probability for AO patients **(Fig 3)**. The nomogram clearly and precisely showed the probability of involvement of each variable. For example, in comparing **Table 3** with **Fig 3**, the higher magnitude odds ratio correlated with the larger point allocation on each of the variables. Therefore, age contributed most to prognosis, followed by surgical treatment, presence of multiple primary malignancies, and marital status. After summing up the total scores of each predictor, the corresponding survival probability of each patient can be obtained.

## Discussion

In the current literature, most studies investigating the detailed clinicopathological features of AO are small case series due to its low incidence and prevalence. The research evaluating prognostic factors influencing the OS of OA is lacking. Our series of 1899 AO patients is the largest study to date. According to previous studies, oligodendroglioma is frequently encountered at age 40 to 60 years, with an average age at diagnosis of approximately 45 years.[1] In our study, the mean age at diagnosis was 49.2 years, slightly older. AO occurs in both men and women, but is slightly more common in men, with the men to women ratio ranging from 1.1 to 2.0.[2] Concordant with the literature, we observed a higher prevalence in male patients with a men to women ratio of 1.3. We also found that the majority of patients were white and married. The overall mean survival time of AO was 56.0 months in this study, with 1-, 3-, 5- and 10-year survival rates of 78.7%, 60%, 50.2%, and 36.2%, respectively. Two previous studies have reported 5-year survival rates of 49.38% and 53.8% [10,11], similar to ours.

Like many other cancers, age at diagnosis has been recognized as a significant prognostic factor for AO.[12] A convincing explanation is that the natural history of glioma changes with increasing age, such as higher rate of proliferation, malignant transformation and larger tumor volume.[12,13] Age at diagnosis was also found to be a significant predictor for survival of AO in our study. Younger patients at diagnosis had better prognosis. In this study, age under 52 years was defined as “younger age”. This approximates prior research defining “younger age” as 50 years old.[10]

In addition to age, marital status has been increasingly recognized as a prognostic factor for cancer. In 1987, Goodwin et al. revealed that marriage was strongly positively correlated with OS of patients with cancer.[14] They studied 27779 patients diagnosed with epithelial cancer and found that unmarried status independently predicted a worse prognosis, with a relative hazard ratio of 1.23.[14] Similarly, Abern et al. demonstrated that married patients with testicular cancer had a higher cancer-specific OS.[15] Our research is the first study to show that single marital status independently predicted worse prognosis in AO. A conceivable explanation is that single patients have less family support and care and may also have poorer compliance with medical treatment. This conclusion emphasizes the importance of supportive nursing and treatment compliance in cancer therapy.

The prognosis of coexistence of malignant tumors of different histologic types is unclear at present. Our research is the first study to find that coexistence of AO and other malignancy predicts shorter survival in AO patients. The underlying pathological mechanism is unclear. Further experimental models and clinical studies of the pathophysiology and prognosis of coexisting malignant tumors are warranted.

Surgery has been well-known as the most crucial treatment for oligodendroglioma.[1] Our study also revealed that surgery was a prognostic factor for AO. Surgery promotes alleviating tumor mass effect and improving neurologic symptoms, and available evidence has shown that a more complete resection of oligodendroglioma improves prognosis.[1] Unfortunately, 12.7% of the surgical records were unclear in our study. Of the remaining, only one case received tumor destruction whereas the others received tumor resection, without detailed information about grade of resection. This limited our ability to further evaluate the association between type and extent of surgery and OS in AO patients.

A nomogram is a valid and crucial tool assisting medical decision-making.[16] It is a visualization of a statistical prediction model that provides survival probability[17] and can provide a quantitative prognosis to help patients more easily understand their condition. In addition, it may assist doctors in dealing with difficult diseases where no guidelines exist. In a nomogram, each predictor is associated with corresponding “points”. We can easily sum up all corresponding “points” to get a “total points”, which correlates to a relevant survival probability. A nomogram providing survival probability is constructed in this study to help both doctors and patients understand the disease easily. Using our nomogram is very simple. For example, if a 40-year-old (40 points) married (0 point) man presented with AO as his only malignant primary tumor (0 point) and received surgery (0 point), the “total points” of this patient is 40 and the corresponding 5- and 10- year survival probabilities would be 67% and 52%, respectively. Consider another example in which a 30-year-old (30 points) single (6 points) woman with AO as her second malignant primary tumor (10 points) did not receive surgery (14 points). The “total points” of this patient is 60 and the corresponding 5-and 10- year survival probabilities would be 41% and 25%, respectively.

Our study suffered several limitations. First, it is clear that interobserver variability exists in the histopathological diagnosis of diffuse glioma and that tumors with a similar microscopic appearance may have different clinical outcomes.[18] Currently, oligodendroglioma is increasingly defined by genetic abnormalities, such as 1p/19q-codeletion and mutations in the isocitrate dehydrogenase gene (IDH).[19] The presence of IDH1 mutations may improve survival in patients with glioma.[20] Also, 1p/19q-codeletion may also associate with a better prognosis in patients with gliomas.[21] While the SEER database does not currently contain this relevant genetic information, this shortcoming may be alleviated somewhat by the high degree of concordance (approximately 80%) between these genetic events and the histologic diagnosis of oligodendrogliomas.[21,22] Though the status of genetic examination in AO is improving, it is unavailable in many hospitals at present and many doctors are unable to utilize it for providing prognosis. However, all the factors included in our nomogram are easily obtained. It can help both doctors and patients understand the disease better. Another limitation in our study is the lack of data on radiotherapy and chemotherapy, which may play a very important role in patient survival. We are unable to clarify the effect of potential advances in radiotherapy and chemotherapy. In addition, other relevant factors, such as biological and immune factors, that may influence the prognosis of AO are not recorded in the SEER database, precluding further stratification of the dataset utilizing these factors. Finally, missing data and selection bias are unavoidable due to the retrospective design of our study. Nevertheless, this study to explore the association between clinical factors and survival outcome in AO patients is the largest one to date.

## Conclusion

In this study, we analyzed a population-based dataset to investigate the demographic characteristics and prognostic factors of a rare disease. We demonstrated that older age, single marital status, and presence of multiple primary malignancies were independently associated with worse survival outcome, whereas surgery was associated with prolonged survival of patients with AO.

## Supporting information

S1 File All relevant data. (XLSX)

S2 File STROBE statement. (DOCX)
